# Adverse Outcome Pathways (AOPs) Oriented Approach to Assess In Vitro Hazard of Silica and Lignin Nanomaterials Derived from Biomass Residues

**DOI:** 10.3390/nano15070549

**Published:** 2025-04-04

**Authors:** Rossella Daniela Bengalli, Maurizio Gualtieri, Mariana Ornelas, Tzanko Tzanov, Paride Mantecca

**Affiliations:** 1 POLARIS Research Center, Department of Earth and Environmental Sciences, University of Milano-Bicocca, Piazza della Scienza 1, 20126 Milan, Italy; paride.mantecca@unimib.it; 2 Interuniversity Center for the Promotion of the 3Rs Principles in Teaching and Research, 56122 Pisa, Italy; 3 CeNTI-Centre for Nanotechnology and Advanced Materials, Rua Fernando Mesquita 2785, 4760-034 Vila Nova de Famalicão, Portugal; 4 Group of Molecular and Industrial Biotechnology, Universitat Politècnica de Catalunya, 08222 Terrassa, Spain; tzanko.tzanov@upc.edu

**Keywords:** adverse outcome pathways, bio-based nanomaterials, lignin nanoparticles, silica nanoparticles, nanotoxicity, polyurethane foams, safe-and-sustainable-by-design

## Abstract

Bio-based nanomaterials (B-NMs), such as silica oxide (SiO_2_)- and lignin (Lig)- based nanoparticles (NPs) derived from biomass waste, have gained attention in the last few years in the view of promoting the sustainability principles in several applications. However, scarce data are available about their safety. Thus, a hazard-testing strategy was designed considering as a reference the safe-and-sustainable-by-design (SSbD) framework for chemicals and materials, prioritizing the use of new approach methodologies (NAMs), such as in vitro and adverse outcome pathways (AOPs) approaches, for generating data about the potential hazard of B-NMs. Literature research was performed to identify the adverse outcomes (AOs) related to the selected B-NMs. All the AOPs investigated shared at least oxidative stress, inflammation and cytotoxicity as key events (KEs) that were investigated in lung and immune cells. The tested B-NMs resulted either non-toxic or moderately toxic towards human cells, validating their biocompatibility when compared to reference NMs of similar composition, but not of bio-origin. However, attention should be given to possible AOs deriving after specific functionalization of the B-NMs. Considering the lack of knowledge in this field, the studies performed represent a step forward in the state of the art of the safety assessment of B-NMs.

## 1. Introduction

Bio-based nanomaterials (B-NMs) derived from biological sources, such as biomass waste from agricultural activities, has gained a lot of attention in the last few years in view of promoting safe and sustainable new technologies [1]. The development of engineered nanoparticles (NPs) by using renewable resources is an example of sustainability and circularity addressing two important environmental issues: (1) the scarcity of natural materials’ supply and (2) the use of residual biomass for added value applications. Among the different B-NMs produced in recent years, bio-based silica NPs (SiNPs) and lignin NPs (LigNPs), derived respectively from cereal husk ashes and lignocellulosic by-products from the paper industry, are widely used in biomedical, agricultural, biotechnological and environmental remediation applications [2,3], thanks also to intrinsic or enhanced functionalities, such as antimicrobial and antifungal [4,5,6,7]. These B-NMs also gained attention for inclusion as (nano)fillers in “greener” polyurethane (PU) composite foams with improved functionalities used in construction, automotive, furniture and bedding industries [8,9,10]. The mechanical and thermal properties of petroleum-based composites are well characterized, as well as their safety profiles, and they ensure the quality criteria requested by the market. The exploration of more suitable waste bio-based materials for synthesizing both biopolyols and bio-based nanofillers can effectively reduce the dependency on petroleum-based polyols. However, both the mechanical, thermal and safety profiles of the new PU are under characterization and further data need to be collected before their placement in the market.

Regarding bio-based nanofillers, in particular, SiNPs could be synthetized from agro-residues like rice husk, a major agricultural waste derived from the rice milling process [11], through acidic recovery process and thermal treatment [2,12,13]. Rice husk contains 65–75% organic constituents (lignin, cellulose and hemicellulose) and 15–20% inorganic substances, such as silica (SiO_2_) [14], making it environmentally friendly and low-cost feedstock of silica [15]. However, the use of rice husk waste is very limited, due to its difficult degradation and high ash content [16]. On the other hand, SiNPs, once obtained, can be easily functionalized with hydroxyl groups, for example, to increase their reactivity in PU foam compositions and endow the foams with hydrophobicity and flame-retardancy. The -OH groups in the surface of the SiNPs make them prone to be easily modified with different functional groups that, in addition to imparting enhanced properties, can also reduce the silica particles affinity for water, thereby facilitating better incorporation into hydrophobic matrices, such as the PU foams [17,18].

Lignin is another widely used renewable material and around 50 million tons of lignin are discharged from the paper industry as a by-product [19]. This biopolymer features phenolic and aliphatic hydroxyl groups that provide antioxidant, antibacterial and anti-UV properties, next to biodegradability and biocompatibility [5,20]. Moreover, lignin in the form of nanoparticles (LigNPs) presents advantages over bulk lignin, such as higher interfacial reactivity in antibacterial and antioxidant nano-enabled materials [21,22]. Both LigNPs and SiNPs functionalized with hydroxyl groups are suitable for inclusion in PU foams representing bio-based polyol nano-constructs that may incorporate into the structure of the foam acting as reinforcing and crosslinking nodes, in addition to imparting water repellence and flame retardance. LigNPs and phenolated lignin (PheLig) NPs have been previously synthetized to evaluate the contribution of the phenolic content to their toxicity and functionality [23].

Despite the great efforts in the synthesis of such B-NMs and their exploitation, encouraged by the industries and by the European Commission, relatively little knowledge is available on their potential impact on human and environmental health. NMs derived from biological sources are often assumed to be non-toxic, but this pre-assumption is not necessarily true [24]. Noteworthy, the large-scale production and use of B-NMs have increased the risk of human exposure through inhalation in occupational settings and in the environment, while their health effects remain unclear. The hazard assessment of novel chemicals and products, including NMs, needs to be performed at different stages of NMs’ life cycle, in accordance to the Safe and Sustainable by Design (SSbD) framework [25], before their upscaling, to increase their market potential without any uncertainties about their safety. Natural and engineered SiNPs are known to cause silicosis in exposed humans and animals and to induce apoptosis and DNA damage in lung cells, liver fibrosis and immunotoxicity [26]. Other response related to SiNPs exposure are associated to epithelial-to-mesenchymal transition and autophagy [27]. These hazardous effects are mostly related to crystalline SiNPs, but it has been shown that amorphous SiNPs are also potentially toxic once inhaled and they can induce carcinogenicity in lung cells [28]. Furthermore, an adverse outcome pathway (AOP) has been proposed for amorphous SiNPs, the AOP 481 “respiratory dysfunction and diseases” [29]. Briefly, an AOP is a conceptual framework that describes a sequence of causally linked events (key events—KEs) at different levels of biological organization, triggered by exposure to a stressor (like a chemical or NM), that lead to an adverse outcome (AO) on health or ecotoxicological relevant species [30]. Regarding LigNPs, little is known about their toxicity after exposure through inhalation, since they have been reported for wound healing purposes and as drug carriers [3], showing biocompatibility in different in vitro and in vivo models [23,31,32,33]. In addition, the methodologies that should be adopted for assessing the potential hazard of B-NMs towards human health and the environment are still not yet defined. The main objective of this work is to test new bio-based NMs, namely SiNPs and LigNPs, considering the possible relevance with putative or future AOPs. After NPs characterization, the potential adverse effects induced by B-NMs were studied by new approach methodologies (NAMs) such as AOP-oriented testing and in vitro biological models. The biological responses induced by pristine SiNPs derived from rice husk were compared to the response from commercial pyrogenic amorphous SiNPs, while pristine LigNPs and phenolated lignin PheLigNPs were investigated by in vitro models representative of the lung (A549 cells) and of the immune system (THP-1 cells). The biological endpoints, in terms of cytotoxicity, oxidative stress and inflammation were evaluated based on AOPs literature research. Tests were also performed in a co-culture model, composed by A549 lung cells and differentiated THP-1 macrophages, which is more representative of the alveolar space. The obtained results are a useful tool for the first-tier screening of the toxicity profiles of the large and growing amount of B-NMs that are going to be developed in the next few years.

## 2. Material and Methods

### 2.1. Chemicals

For the extraction of SiNPs from rice husk (SiO_2_-RHSK), rice husk (RH) was supplied by Arrozeiras Mundiarroz, S.A. (Coruche, Portugal) and hydrochloric acid (37% *v*/*v*) was provided by Thermo Fischer Scientific (Oeiras, Portugal). Industrial SiNPs (SiO_2_-Aerosil) were purchased by Evonik (Aerosil^®^200, Evonik Industries AG, Essen, Germany) and produced by flame synthesis. For the production of LigNPs and PheLigNPs, the protocol reported by Bragato et al. [23] was used.

Cell culture medium (Opti-MEM, Gibco, Life Technologies, Monza, Italy), foetal bovine serum (FBS, Gibco), 2′,7′-dichlorofluorescein diacetate (H_2_DCFDA), Alamar Blue^®^ reagent, ELISA buffer kit and matched antibody pairs for IL-8, IL-1β, IL-6 were purchased by Life Technologies (Monza, Italy). Dulbecco Modified Eagle Medium (DMEM) high glucose and dimethyl sulfoxide (DMSO) and penicillin/Streptomycin (Pen/Strep) were provided by Euroclone (Pero, Italy). Sodium bicarbonate (NaHCO_3_), calcium sulphate dyhydrate (CaSO_4_xH_2_O), Lipopolysaccharides (LPS) from *Escherichia coli O111:B4*, Trypsin-EDTA 1X and Accutase were purchased from Sigma Aldrich (Milan, Italy). Human alveolar epithelial cell line A549 (ATCC^®^ CCL-185™) and THP-1 cell (ATCC^®^-TIB-202™) line were provided by the American Type Culture Collection (ATCC, Manassas, VA, USA).

### 2.2. Literature Search and AOP-Wiki Database Examination

The literature search and the examination of AOP-Wiki database for the selected B-NMs mechanisms of toxicity was organized following the work of Kose et al. [34]. We divided our search into two stages: AOP-Wiki database review and literature review using PubMed.

First, we used the AOP-Wiki to identify the AO potentially related to the B-NMs. The AOP-Wiki is the central database for capturing and reviewing AOP knowledge. As prototypical stressors, we put as inputs the following keywords: nanoparticles, silica nanoparticles, insoluble nanosized particles.

Secondly, potential AOPs developed to describe NP toxicity but not included in the AOP-Wiki database were identified in PubMed. The search pattern for terms and the respective research results were as follows: (1) nanoparticles [All Field] AND adverse [All Field] AND outcome [All Field] AND pathways (175 documents); (2) silica [All Field] AND nanoparticles [All Field] AND adverse [All Field] AND outcome [All Field] AND pathways [All Field] (eight documents); (3) SiO_2_ [All Field] AND nanoparticles [All Field] AND adverse [All Field] AND outcome [All Field] AND pathways [All Field] (four documents results); (4) lignin AND nanoparticles AND adverse AND outcome AND pathways (no documents found); (5) insoluble AND nanoparticles AND adverse AND outcome AND pathways (three documents). The articles were then screened considering the effects of SiNPs and LigNPs on in vitro models of the lung and immune systems as target, focusing on respiratory and circulatory diseases as final adverse outcome. Two articles were found that associated the effects of SiNPs on AO related to cardiovascular and respiratory health [35,36].

### 2.3. SiNPs and LigNPs: Synthesis, Preparation of Suspensions and Physico-Chemical Characterization

For the in vitro testing, the following NPs were used:

SiNPs: commercial SiO_2_-Aerosil NPs (Aerosil®200) from pyrogenic synthesis and bio-based silica NPs from rice husk (SiO_2_-RHSK). Moreover, alternatives of the SiO_2_-RHSK NPs modified with the addition of functional groups were tested. Detailed information about the modified silica forms is reported in the Supplementary Materials.

LigNPs: sonochemically nano-formulated pristine lignin (LigNPs) and an enzymatically phenolated one (PheLigNPs).

#### 2.3.1. Synthesis of SiNPs and LigNPs

SiO_2_ NPs extracted from rice husk (SiO_2_-RHSK) were obtained by an acidic digestion followed by a calcination process [37]. Briefly, 10% wt. HCl was added to RH (previously milled to 1.5 mm) and the digestion occurred for 2 h at 60 °C. SiO_2_-RHSK particles were recovered after a calcination step at 700 °C for 3.5 h (ramp: 4.5 °C/min). The further modification of SiO_2_-RHSK with phytic acid (PA) and 3-(trimethoxysilyl)-propyldimethyloctadecylammonium chloride (QAS) (SiO_2_-RHSK + PA + QAS NPs) is described in the Supplementary Materials.

Briefly, bio-based SiNPs from rice husk (SiO_2_-RHSK) were modified by introducing OH groups to synthetize SiO_2_-RHSK-OH, for increased reactivity and hydrophobicity, in which it was further functionalized by the addition of PA and QAS to synthetized produce SiO_2_-RHSK + PA + QAS NPs for flame retardancy and antimicrobial properties. Moreover, SiO_2_-RHSK NPs were also grafted with polyol (BI-3521), called PM134.

The synthesis methods of LigNPs and PheLigNPs are reported in [23,38].

#### 2.3.2. Preparation of SiNPs and LigNPs Suspensions

For the in vitro testing, the following NP suspensions were prepared:

SiO_2_-Aerosil and SiO_2_-RHSK were weighed by using a micro-balance (Sartorius, Goettingen, Germany) in sterile conditions, under a laminar flow hood, suspended in sterile ultrapure water to reach a concentration of 1 mg/mL (stock solution). Then, suspensions were sonicated with the sonicator Bandelin Sonopuls (Berlin, Germany) equipped with a tip probe with the setting of 20% amplitude, 1 s pulse, 1 s stop, for 10 min to reach an energy of 3 kJ/s. Suspensions were stored at 4 °C temperature, for a period no longer than 30 days. Different stock suspensions were prepared and independently tested during the experiments. LigNPs and PheLigNPs suspensions were prepared as previously by Bragato and co-workers [23]. The suspensions were diluted in sterile milli-Q water to reach the concentration of 1 mg/mL and then directed diluted in cell culture media at the desired concentrations for the in vitro treatments.

#### 2.3.3. Physico-Chemical (p-Chem) Characterization of SiNPs and LigNPs

SiNPs (SiO_2_-Aerosil and SiO_2_-RHSK) and LigNPs (LigNPs and PheLigNPs) suspensions were characterized by transmission electron microscopy (TEM) (Jeol JEM 2100Plus, JEOL, Tokyo, Japan) for their morphology and shape, while Dynamic Light Scattering (DLS) and Electrophoretic Light Scattering (ELS) (Zetasizer Nano ZS90 Malvern Ltd., Warwickshire, UK) were used to assess their agglomeration state, size distribution (z-average) and surface charges (ζ-potential).

Moreover, the SiO_2_-RHSK NPs were characterized through thermogravimetric (TGA, DG), FTIR, and SEM-EDS analyses (see Supplementary Materials). LigNPs were fully characterized through thermogravimetric analysis (TGA, Hitachi, Tokyo, Japan) and Fourier Transform Infrared Spectroscopy (FTIR, Perkin Elmer, Beaconsfield, United Kingdom), as reported in previous papers [23,39].

For TEM analysis, an NPs suspension of 100 µg/mL was prepared in milli-Q (mQ) water from the stock solution and a drop (10 µL) was dropped on a coated 300-mesh copper grid. The grids were let to dry overnight and then observed with a TEM, operating at an acceleration voltage of 200 kV and equipped with an 8 MPx complementary metal oxide semiconductor (CMOS) Gatan Rio9 (Gatan, Pleasanton, CA, USA) digital camera.

For DLS analysis, NPs (final concentrations 100 µg/mL) were dispersed in different media: (1) milli-Q water and (2) cell culture media: DMEM and OptiMEM media supplemented with 1% foetal bovine serum (FBS) and analysed immediately after the preparation of the NPs suspension (time 0, t0) and after 24 h (t24) at 37 °C (temperature of cell maintenance). For ELS analysis, NPs were prepared in mQ water at the concentration of 100 µg/mL/mL and immediately analysed for their ζ-potential with the instrument Zetasizer Nano ZS90 (Malvern Ltd., Warwickshire, UK).

### 2.4. B-NMs In Vitro Hazard Assessment in Cell Monocultures

#### 2.4.1. Cell Maintenance and Treatments of Monocultures

Human alveolar cells A549 and THP-1 immune cells were cultivated in monocultures using DMEM and OptiMEM medium, respectively, both supplemented with 10% foetal bovine serum (FBS) and 100 U/mL penicillin/streptomycin and maintained at 37 °C, 5% CO_2_. For treatments, cells were seeded in 6-wells (A549 cells, 2.5 × 10^5^ cells/well) and 12-wells (THP-1 cells, 2 × 10^5^ cells/well) and treated with increasing concentrations of B-NMs (0, 1, 10, 50, 100 µg/mL) for 24 h. For the treatments, B-NMs were dispersed in cell culture medium with 1% FBS. Untreated cells were used as negative controls. LPS was used (10 µg/mL) as the positive control for the induction of inflammatory response, while H_2_O_2_ (100 µM) was used for 90 min as the positive control for the induction of ROS.

#### 2.4.2. Cell Viability (MTT, Alamar Blue)

After exposure to SiNPs and LigNPs, cell viability was assessed with a metabolic test Alamar^®^ Blue reagent (Life Technologies, Monza, Italy). For A549, after 24 h of exposure to the B-NMs, supernatants were collected, centrifuged at 1200 rpm for 6 min and then stored at −80 °C for ELISA analyses. Cells were then washed with PSB 1X and incubated with fresh medium containing 10% of Alamar Blue reagent. After 2–4 h of incubation at 37 °C, 5% CO_2_, the products of reaction were transferred into a 96-well black multiplate (200 µL for well) and then the fluorescence was read with the TECAN Infinite M200 Pro microplate reader (TECAN, Männedorf, Switzerland) at an excitation wavelength of 560 nm, an emission wavelength of 590 nm and a gain of 82. The viability was expressed as relative percentage variation over the negative control (untreated cells) (100% cell viability).

Briefly, after exposure to B-NMs cells, the THP-1 cells were collected in a 1.5 mL vial and centrifuged for 6 min at 1200 rpm. The supernatants were collected and stored at −80 °C for the ELISA test. The resulting pellets were resuspended with cell medium containing 10% of Alamar Blue reagent. Cells were replaced in the multiwell and incubated at 37 °C for 2–3 h. After incubation, the cells were collected, centrifuged for 6 min at 1050 rpm and then supernatants (200 µL) were transferred into a 96-well black plate. The fluorescence was read as previously described.

An MTT test was performed on A549 cells exposed to SiNPs modification (described in the Supplementary Materials). After the 24 h exposure to SiNPs and supernatants collection, cells were rinsed with phosphate buffer saline (PBS) and then a solution of 0.3 mg/mL of MTT, prepared in cell culture medium supplemented with 10% FBS, was added to the cells. Cells were incubated for 3 h at 37 °C, 5% CO_2_ for the conversion by metabolic active cells of the tetrazolium salt in formazan crystals. Then, supernatants were removed by aspiration and the precipitated crystals were solubilized using 1 mL of DMSO for each well and left for 10 min with continual shaking, before transferring 200 µL from each well into a 96-well transparent plate in triplicate. The absorbance was measured at 570 nm with a reference of 690 nm with a TECAN Infinite M200 Pro microplate reader (TECAN, Männedorf, Switzerland). The viability is expressed as the percent control ratio. Data were presented as mean ± SEM of at least five independent experiments.

#### 2.4.3. Intracellular Reactive Oxygen Species Detection

The detection of intracellular reactive oxygen species (ROS) was performed in A549 and THP-1 after exposure for 90 min to 10 and 50 µg/mL to the different NPs, using the probe H_2_DCFDA (final concentration 10 μM). A549 cells (9 × 10^5^ cells/well) and THP-1 cells (20 × 10^5^ cells/well) were seeded in 12-well plates and loaded with H_2_DCFDA in PBS for 20 min at 37 °C. After the loading with the probe, cells were washed twice with PBS, incubated for 90 min with SiNPs or LigNPs, then washed with PBS, and collected by centrifugation at 1200 rpm for 6 min. Pellets were resuspended in PBS and cell suspensions were then analysed by flow cytometry (CytoFLEX, Beckman Coulter, Milan, Italy) with excitation and emission settings of 488 nm and 525 nm, respectively. Untreated cells were used as the negative control, H_2_O_2_ (100 µM) was used as the positive control, while the fluorescence intensity of cells not loaded with the probe was subtracted to the respective treated cells, also to avoid interference with the potential auto-fluorescence of NPs.

#### 2.4.4. Release of Inflammatory Mediators (IL-8, IL-6 and IL-1β)

LPS at the concentration of 10 µg/mL was used as the positive control for the inflammatory response in parallel with B-NMs treatments, for both A549 and THP-1 cells. After the 24 h treatment, supernatants were collected, centrifuged at 1200 rpm for 6 min and kept at −80 °C until analysis. The release of interleukin-8 (IL-8), interleukin-6 (IL-6) and interleukin-1β (IL-1β) was assessed with ELISA (Life Technologies, Monza, Italy) matched antibody kits following the manufacturer’s instructions. The sample absorbance was measured by a multiplate reader (Infinite 200 Pro, TECAN) at the wavelength of 450 nm; the concentration of interleukins was calculated based on standard curves and data were shown as pg/mL. Untreated cells were considered as the negative control.

### 2.5. B-NMs In Vitro Hazard Assessment in Cell Co-Cultures

#### 2.5.1. Co-Culture Set-Up and Quasi-ALI Exposure

**Figure 1:**
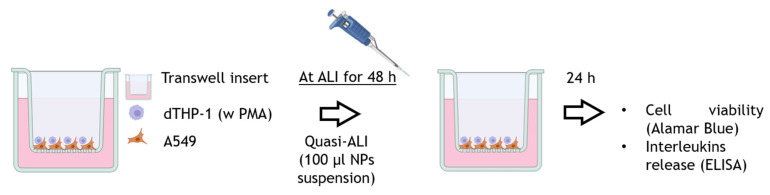
Schematic representation of the in vitro co-culture model and set up of the quasi-ALI exposure to B-NMs.

The different NPs analysed within the project were used in a co-culture in vitro model, composed of alveolar pulmonary cells (A549) and THP-1–derived macrophages (dTHP-1) cultivated on Transwell™ inserts, following the protocol of Motta and co-workers [40]. This co-culture model allows to better analyse NPs’ effect, considering the relevance of macrophages in the inflammatory response. Briefly, A549 cells were seeded on Transwell™ inserts (pore 1 µm, diameter 1.12 cm^2^, cellQART, SABEU GmbH & Co. KG, Northeim, Germany) at a density of 5 × 10^4^ cells/insert and allowed to grow for 48 h in OptiMEM medium supplemented with 10% of FBS. THP-1 cells were differentiated into macrophages by using 20 ng/mL of Phorbol 12-myristate 13-acetate (PMA) for 24 h and then cultivated for further 24 h in fresh OptiMEM medium supplemented with 10% FBS for recovery. Then, differentiated macrophages were detached from the flask with Accutase, centrifuged for 6 min at 1050 rpm and then seeded directly on the inserts, in direct contact with A549 cells. The ratio among dTHP-1 and A549 cells was 1:10. After 4 h from the seeding of the dTHP-1 onto A549 cells, the co-culture was put at the ALI by removing the medium from the apical side of the inserts, and in the basal side were added 0.6 mL of fresh OptiMEM medium. After 24–48 h at ALI, the co-cultures were considered differentiated, as previously reported [40], and they were exposed to B-NMs at quasi-ALI by adding 100 µL of B-NMs suspension directly in the apical compartment [41]. After 24 h of exposure to either SiNPs and LigNPs at the concentrations of 5 and 10 µg/cm^2^ (corresponding to 50 and 100 µg/mL used in the monoculture models), cell viability and pro-inflammatory mediators release (IL-1β, IL-6 and IL-8) were evaluated. Figure 1 shows a schematic representation of the set-up of the in vitro co-culture model and of the quasi-ALI exposure.

#### 2.5.2. Cell Viability and Inflammatory Responses of Co-Culture Models

The cytotoxicity of SiNPs and LigNPs on co-cultures was evaluated through the Alamar Blue test as previously described. For the detection of cytokines released from the co-culture models after quasi-ALI exposure, the undernatants were collected from the basal compartment of the inserts, centrifuged at 1200 rpm for 6 min and stored at −80 °C until ELISA tests were performed.

### 2.6. Statistical Analysis

The data represent the mean ± standard error of the mean (SEM) of at least three independent experiments (n ≥ 3). For cell viability tests, a minimum of five independent experiments was considered (n = 5). Statistical analyses were performed using Sigma Stat 3.2, using one-way ANOVA and Bonferroni’s post hoc analysis, if not elsewhere specified in the figure captions. GraphPrism was used for the making the graphs. Values of *p* < 0.05 were considered statistically significant.

## 3. Results

### 3.1. Literature and AOP-Wiki Research for the Definition of the Biological Endpoints

**Figure A1:**
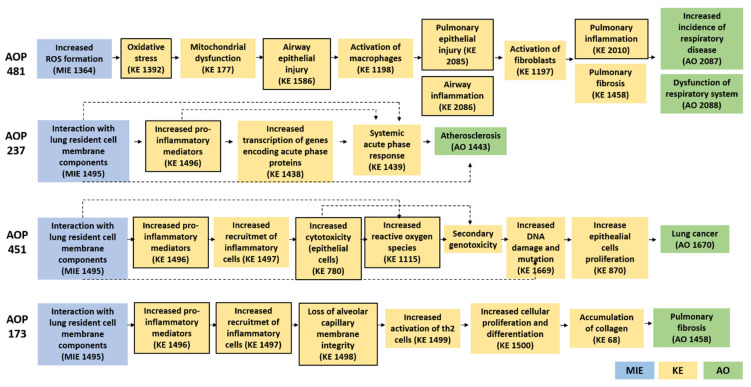
AOP-Wiki results: AOPs related to B-NMs (NPs, SiNPs and LigNPs (insoluble NPs)) as a potential stressor. The AOPs were analysed in terms of their molecular initiating events (MIE, blue boxes), key events (KE, yellow boxes) and adverse outcomes (AO, green boxes). This led to the identification of pathways and KEs that are shared among these AOPs (black highlighted yellow boxes), focusing on oxidative stress, inflammation and loss of alveolar function (cytotoxicity). These pathways served as the basis of our AOP-oriented testing strategy.

A list of the AOPs for the selected B-NMs identified through the AOP-Wiki database is reported in Appendix A, Table A1. The literature and AOP-Wiki search were not restricted to the impact of B-NMs on lung and immune cells, but also included impact on liver toxicity and inflammation in general. We focused our attention on AOPs of relevance for respiratory and circulatory diseases related to NMs—due to the cell lines selected for the in vitro tests—and their applicability in identifying key events (KEs) of toxicity for targeted in vitro assays development. One AOP was identified showing that NPs are a stressor (Appendix A, Figure A1) related to lung cancer (AOP 451). The interaction of NPs with the resident cell membrane component is the molecular initiating event (MIE) that leads to the AO (lung cancer). The pathway comprises an increased secretion of pro-inflammatory mediators and recruitment of inflammatory cells, with consequent increased cytotoxicity of epithelial cells, increased ROS formation that finally leads to secondary genotoxicity (DNA damage and mutation) and augmented cell proliferation [42]. The AOP 481 is related to amorphous SiNPs. The ROS-mediated oxidative stress induced by these NPs increases airways epithelial damage and inflammation, causing an increased incidence of respiratory dysfunction and diseases. Insoluble nanosized particles, such as LigNPs, are related to the AOP 237, in which the substance interaction with lung resident cell membrane components increases the secretion of proinflammatory mediators and the transcription of genes encoding acute phase proteins and, therefore, systemic acute phase responses that could cause atherosclerosis [43]. Moreover, the AOP 173 substance interaction with the pulmonary resident cell membrane components (MIE) leading to pulmonary fibrosis could also be related to the exposure to silica NPs and other NMs (i.e., carbon nanotubes) [44,45]. According to this AOP, the MIE triggers the increased secretion of pro-inflammatory and pro-fibrotic mediators and of ROS levels, loss of alveolar membrane integrity, activation of Th-2 helper, proliferation of fibro/myofibroblasts and extracellular matrix (ECM), causing lung fibrosis and increased mortality. Although AOP 173 was not found in the AOP-Wiki as directly related to silica and insoluble NPs, there are evidences from human and animal studies that the chronic exposure to SiNPs lead to the development of silicosis in concordance with the AOP presented [46].

The networks of the AOPs associated to the selected stressors are reported in Figure A1, showing the main key events (KEs) involved and the resulting adverse outcome (AO). Based on the analysis of these AOPs, three mechanisms can be identified (black bolded yellow boxes in Figure A1, Appendix A), which are common to the described adverse outcomes. These mechanisms are related to cell oxidative balance disturbance (ROS production and accumulation, oxidative stress, mitochondrial damage), inflammatory responses (release of cytokines) and loss of alveolar function (cytotoxicity). Accordingly, we focused the experimental assessment of B-NMs’ toxicity towards lung and immune cells by evaluating the cytotoxicity, ROS accumulation and release of inflammatory mediators triggered by SiNPs and LigNPs.

### 3.2. NPs Characterization

**Figure 2:**
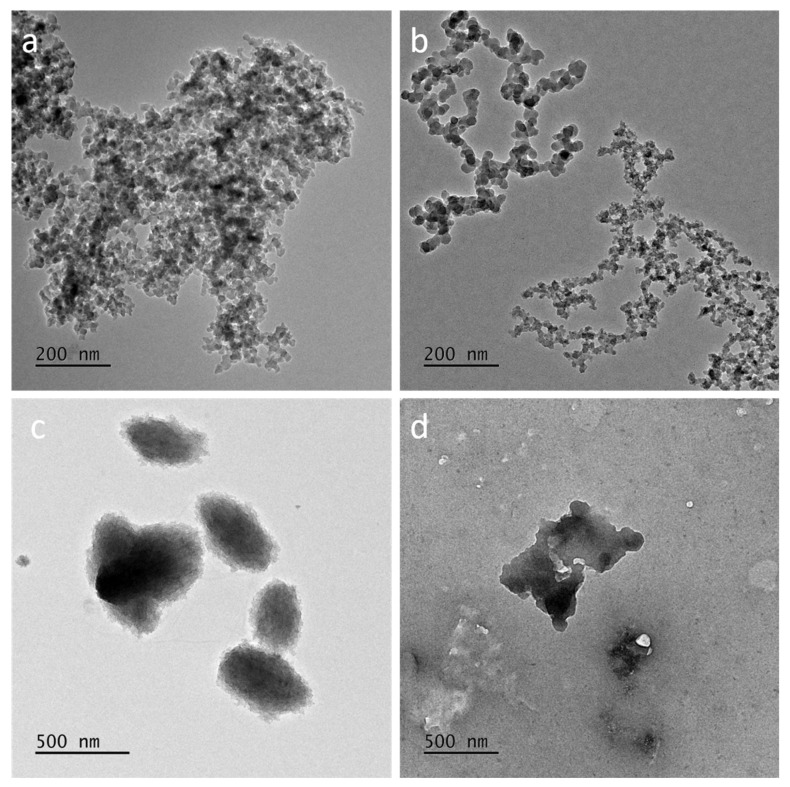
TEM analysis of B-NMs. TEM images of SiO_2_-Aerosil (**a**), SiO_2_-RHSK (**b**), Lig (**c**) and PheLig (**d**) NPs in mQ water. Scale bars: 200 nm (**a**,**b**) and 500 nm (**c**,**d**).

TEM images of SiNPs and LigNPs resuspended in milli-Q water were recorded (Figure 2). SiO_2_-Aerosil NPs (Figure 2a) are pyrogenic amorphous spheroidal NPs with a core size of around 50 nm that form agglomerates of hundreds of nanometres. SiO_2_-RHSK NPs (Figure 2b) have a primary size of around 30 nm that form agglomerates with a branched shape. LigNPs (Figure 2c) have an oval shape with a dimension below 500 nm that do not form bigger agglomerates, while PheLig NPs (Figure 2d) have an irregular shape and form agglomerates of particles of around 500 nm.

Data from DLS (Table 1) showed that the SiO_2-_Aerosil have a hydrodynamic diameter of 246.7 ± 40.5 nm and a PdI of 0.225 when dispersed in milli-Q water, while they form bigger agglomerates once dispersed in cell culture medium (z-average = 625.5 ± 344.7 nm in DMEM 1% FBS and 702.1 ± 261.6 nm in OptiMEM 1% FBS). SiO_2_-RHSK NPs form bigger agglomerates also in milli-Q (507. 9 ± 104.8 nm), with a PdI of 0.693 ± 0.037.

Also, in this case, in cell culture media, both z-average (1022.7 ± 510.04 nm in DMEM 1% and 1359.1 ± 821.5 nm in OptiMEM 1%) and PdI (0.888 ± 0.096 and 0.925 ± 0.110 in DMEM and OptiMEM 1% FBS, respectively) values increase. Lower PdI values, indicating more homogenous agglomerate in term of average hydrodynamic size, were reported for the commercial Aerosil SiNPs dispersed in milli-Q. Both Aerosil and SiO_2_-RHSK NPs have a negative ζ-potential of −21.03 ± 0.62 mV and −22.80 ± 0.14 mV, respectively. Furthermore, after 24 h of incubation in cell culture media, the z-average and PdI values strongly decreased compared to t0 samples, probably due to sedimentation of bigger agglomerate of SiNPs on the bottom of the cuvette.

Results from thermogravimetric analysis, FTIR, and SEM-EDS of SiNPs are reported in the Supplementary Materials (Supplementary Figure S1). The residual mass observed in the thermogram for SiO_2_-RHSK was higher than 95%, coherent with the inorganic nature composition expected for the synthetized particles. For these particles, the observed weight loss is atttributed to adsorbed moisture. Regarding the FTIR analysis (Supplementary Figure S1c), the spectra for all samples exhibited bands at ca. 800 and 1050 cm^−1^, which can be assigned to the symmetric and asymmetric stretching vibration of Si-O-Si bonds, respectively [47,48]. As expected, similar spectra were obtained for SiO_2_-RHSK and SiO_2_-Aerosil. SEM images for the SiO_2_-RHSK, as well as the corresponding chemical composition determined by EDS, is shown in Supplementary Figure S1d. The SiO_2_ particles recovered from rice husk exhibited a spherical morphology in the range of 40–80 nm. The EDS analysis confirmed the presence of Si and O in the particles (the observed C is a contamination from the carbon tape used to prepare the samples for analysis). SiNPs modified with functional groups were characterized at TEM and through DLS and the results are reported in Supplementary Figure S2 and Table S1. Regarding lignin-based NPs, the z-average values of LigNPs and PheLigNPs (100 µg/mL), in DMEM medium at time 0, are 528.8 ± 2.41 nm and 871.2 ± 107.1 nm, respectively, which are higher compared to the one measured in milli-Q water (355.5 ± 7.3 nm for LigNPs and 525.4 ± 23.29 nm for PheLigNPs). After 24 h, the values of z-average are lower (210.9 ± 4.15 nm for LigNPs and 196.6 ± 4.64 nm for PheLigNPs), probably due to sedimentation of the bigger particles. A similar trend was also observed for both LigNPs in OptiMEM cell culture medium. The PdI values of LigNPs are lower compared to the one observed for SiNPs, suggesting that LigNPs are more monodispersed. LigNPs and PheLigNPs have similar ζ-potential values of −33.2 ± 2.5 mV and −32.7 ± 0.45 mV, respectively.

### 3.3. In Vitro Cellular Responses in Monocultures

#### 3.3.1. Cell Viability

**Figure 3:**
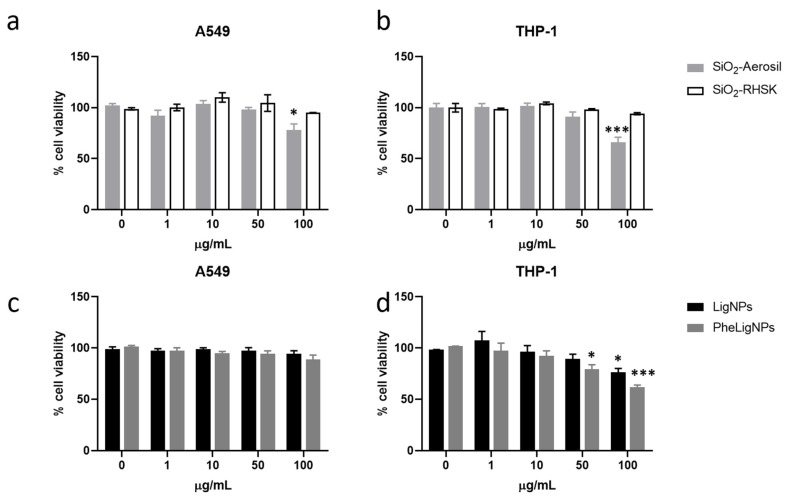
Cell viability of A549 and THP-1 cells exposed to B-NMs. Cell viability of A549 (**a**,**c**) and THP-1 (**b**,**d**) cells by Alamar Blue assays, respectively, after 24 h of exposure to increasing concentrations (1–100 µg/mL) of SiO_2_-Aerosil NPs (grey bars) and SiO_2_-RHSK (white bars) and Lig (black bars) and PheLig (dark grey bars) NPs. Data represents the mean percentage over the control ± SEM of five independent experiments (n = 5). * *p* < 0.05; *** *p* < 0.001 (ANOVA + Bonferroni’s test).

Results show that SiO_2_-Aerosil induced a significant cell viability reduction at the concentration of 100 µg/mL in both A549 and THP-1 cells (Figure 3a,b) of the 22% and 34%, respectively, compared to the negative control. On the contrary, SiO_2_-RHSK NPs did not affect cell viability even at the highest tested concentration. With respect to lignin-based NPs, PheLigNPs induced a slight though not significant reduction in cell viability at the concentration of 100 µg/mL in A549 (Figure 3c); similarly, LigNPs lacked effects on cell viability. In THP-1 cells, PheLigNPs induced a significant reduction in cell viability of 20% with respect to the control sample (untreated cells) already at the concentration of 50 µg/mL (Figure 3d) and of the 39% at the highest tested concentration (100 µg/mL). LigNPs at the concentration of 100 µg/mL also significantly reduced THP-1 cell viability of 23%. Interestingly, no effects were observed in A549 exposed to lignin-based NPs.

As previously mentioned, the additional value of SiO_2_-RHSK NPs is the possibility to add functional groups for enhancing their incorporation in PU foams, while giving them other properties, such as flame retardant or antimicrobial properties. Thus, different modification of SiO_2_-RHSK were further tested for their toxicity on A549 cells. Concerning modified SiO_2_-RHSK (SiO_2_-RHSK + PA + QAS), data on A549 cells showed that the functionalization with PA and QAS increases the cytotoxicity of the NPs, while the addition of polyol did not affect the cell viability of lung cells (Supplementary Materials, Supplementary Figures S3a and S4a).

#### 3.3.2. ROS Formation

**Figure 4:**
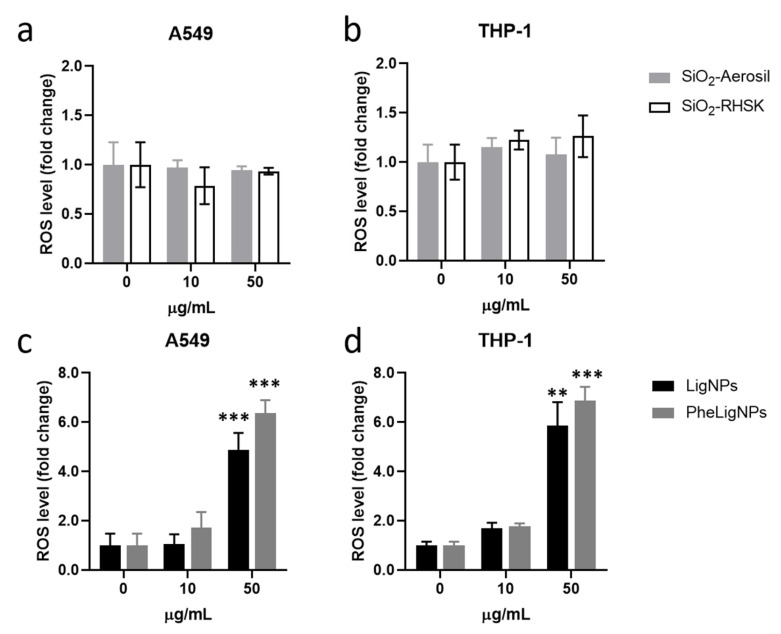
Intracellular ROS level induced by B-NMs. ROS level of A549 (**a**,**c**) and THP-1 (**b**,**d**) cells exposed for 90 min to SiNPs (**a**,**b**) and LigNPs (**c**,**d**). A549 and THP-1 cells were exposed to 0, 10 and 50 µg/mL of either SiO_2_-Aerosil NPs (grey bars), SiO_2_-RHSK (white bars) and Lig (black bars) and PheLig (dark grey bars) NPs data represents the mean of the fold change over the control ± SEM of three independent experiments (n = 3). ** *p* < 0.01; *** *p* < 0.001 (ANOVA + Bonferroni’s test). Statistically different from the control samples (untreated cells).

The capability of silica- and lignin-based NPs to increase the oxidative stress in exposed cells was assessed by the detection of intracellular ROS. Results showed that both SiO_2_-Aerosil and SiO_2_-RHSK NPs did not induce increased levels of ROS in both A549 and THP-1 cells after 90 min of exposure (Figure 4a,b). Moreover, after 24 h of exposure to 50 µg/mL of the different silica-based NPs, SiO_2_-Aerosil NPs were able to induce a significant increase in intracellular ROS formation (Supplementary Materials, Supplementary Figure S3b) of 2.1-fold, while SiO_2_-RHSK NPs did not affect the intracellular ROS levels. Moreover, functionalized SiO_2_-RHSK + PA + QAS also induced significant increased levels of ROS (8.8-fold compared to the control samples) in A549 cells exposed for 24 h (Supplementary Materials, Supplementary Figure S3b).

Results from intracellular ROS measurements in A549 exposed for 90 min to different concentrations of LigNPs and PheLigNPs revealed that these NPs were able to induce a significant 5- and 6-fold increase in ROS levels, respectively, at the highest concentration tested (50 µg/mL) (Figure 4c). Data on THP-1 cells treated with the particles for 90 min showed that PheLigNPs induced a 7-fold increase in ROS production after the exposure to 50 µg/mL, while the LigNPs (50 µg/mL) increased the ROS levels by 5.5-fold compared to untreated cells (negative controls) (Figure 4d). The level of ROS detected in A549 and THP-1 cells after the exposure to the positive control (H_2_O_2_, 100 µM) are reported in the Supplementary Materials (Supplementary Figure S5).

#### 3.3.3. Release of Inflammatory Mediators

**Figure 5:**
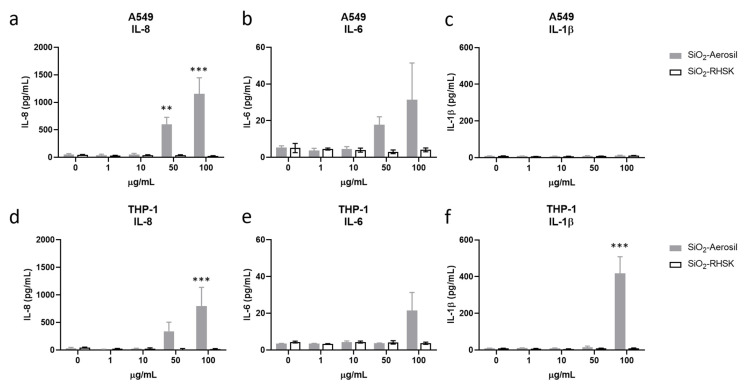
Release of inflammatory mediators after exposure to SiNPs. The release of IL-8 (**a**,**d**), IL-6 (**b**,**e**) and IL-1β (**c**,**f**) was assessed after 24 h of exposure of A549 (**a**–**c**) and THP-1 (**d**–**f**) cells to increasing concentrations (1–100 µg/mL) of SiO_2_-Aerosil NPs (grey bars) and SiO_2_-RHSK (white bars) NPs. Data represents the mean ± SEM of at least three independent experiments (n ≥ 3). ** *p* < 0.01, *** *p* < 0.001 (ANOVA + Bonferroni’s test).

**Figure 6:**
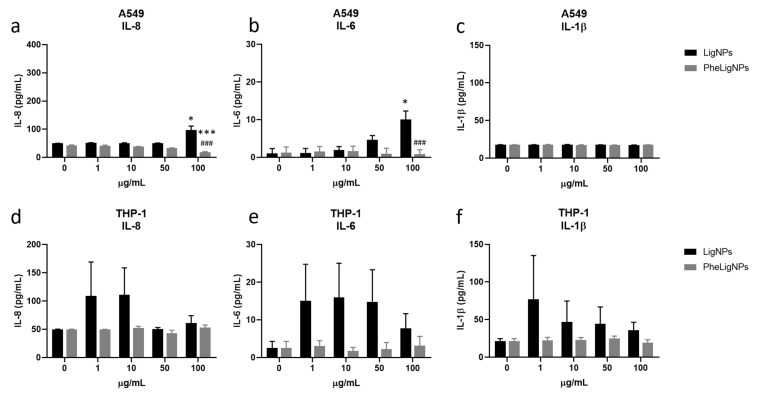
Release of inflammatory mediators after exposure to LigNPs. The release of IL-8 (**a**,**d**), IL-6 (**b**,**e**) and IL-1β (**c**,**f**) was assessed after 24 h of exposure of A549 (**a**–**c**) and THP-1 (**d**–**f**) cells to increasing concentrations (1–100 µg/mL) of LigNPs (black bars) and PheLigNPs (dark grey bars). Data represents the mean ± SEM of at least three independent experiments (n ≥ 3). * *p* < 0.05, *** *p* < 0.001, ^###^ *p* < 0.001. * Statistically different compared to the control sample. ^###^ Statistically different compared to LigNPs (100 µg/mL) (ANOVA + Bonferroni’s test).

The inflammatory response induced by silica- and lignin-based NPs was investigated through the quantification of IL-8, IL-6 and IL-1β released in A549 and THP-1 monoculture after 24 h of exposure to SiNPs (Figure 5) and LigNPs (Figure 6). SiO_2_-Aerosil NPs induced a significant increased release of IL-8 from A549 cells of 598 ± 128 pg/mL with respect to the control samples (51 ± 16 pg/mL), starting from the concentration of 50 µg/mL (Figure 5a), while in THP-1 cells (Figure 5d) a significant increase in IL-8 release was observed at the concentration of exposure of 100 µg/mL (801 ± 336 vs. control 33 ± 14 pg/mL). A slight increase in IL-8 was observed also at 50 µg/mL, although not significant. SiO_2_-RHSK did not affect the release of IL-8 in both A549 and THP-1 cell lines. With regards to IL-6, SiO_2_-Aerosil NPs induced in A549 cells an increase in the protein release, although not significant, at the concentration of 50 and 100 µg/mL (Figure 5b). Increased levels of IL-6 were also observed in the supernatants of THP-1 cells exposed to 100 µg/mL of SiO_2_-Aerosil NPs (21 ± 9 pg/mL vs. control 3.5 ± 0.09 pg/mL) (Figure 5e). The exposure to bio-based SiO_2_-RHSK NPs did not increase the release of the cytokine IL-6 in both cell lines. Moreover, results showed that the release of IL-1β in A549 cells was not induced by the treatment with SiO_2_-Aerosil and SiO_2_-RHSK NPs (Figure 5c) and values are close to the detection limit, while a significant release of this cytokine was observed in THP-1 treated to 100 µg/mL of SiO_2_-Aerosil NPs (418 ± 90 pg/mL vs. control 8.6 ± 1.9 pg/mL) (Figure 5f).

The effects of LigNPs on the inflammatory response showed interesting results. PheLigNPs in fact reduced the release of cytokines in lung cells. The release of IL-8 from A549 cells is significantly increased after the exposure to LigNPs (97 ± 13 pg/mL vs. 49 ± 0.5 pg/mL in the control samples), while IL-8 is reduced after the exposure to PheLigNPs at the concentration of 50 and 100 µg/mL (33 ± 0.3 pg/mL and 18 ± 2 pg/mL, respectively), compared to the control samples (42 ± 1.6 pg/mL). Furthermore, at the highest concentration tested (100 µg/mL), the levels of IL-8 released from A549 is statistically lower when compared to LigNPs released at the same concentration (Figure 6a). Data regarding IL-6 showed that the exposure to LigNPs increases the release of this cytokine at the concentration of 100 µg/mL (10 ± 2 pg/mL), while PheLigNPs did not affect the release of IL-6 (Figure 6b). Finally, both Lig- and PheLigNPs did not modulate the release of IL-1β from lung cells (Figure 6c). Whilst a modulation of the inflammatory response was observed after the exposure of A549 to both LigNPs, in THP-1, no significant variation of the levels of the different ILs tested was observed (Figure 6d–f).

### 3.4. In Vitro Responses in Co-Culture Models

**Figure 7:**
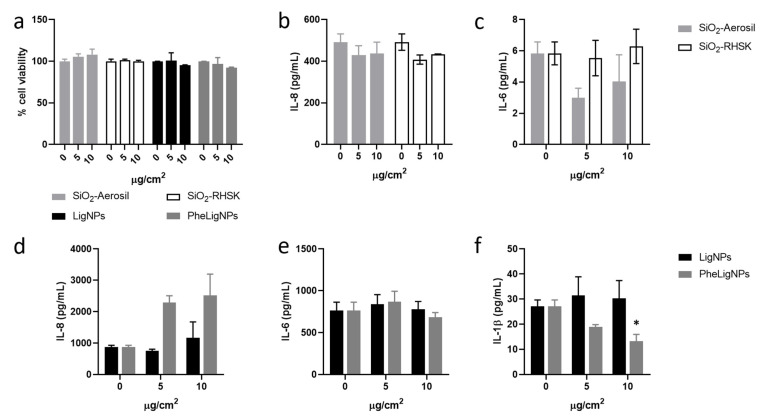
Cell viability and inflammatory responses of the co-culture lung model exposed to B-NMs (SiNPs and LigNPs). (**a**) Cells viability of co-culture models assessed by the Alamar Blue test after 24 h of exposure to increasing concentrations of SiNPs and LigNPs (5 and 10 µg/cm^2^). (**b**,**c**) The release of inflammatory mediators IL-8 (**b**) and IL-6 (**c**) from the co-culture model exposed to SiNPs was assessed after 24 h of exposure to increasing concentrations (5 and 10 µg/cm^2^) of SiO_2_-Aerosil NPs (grey bars) and SiO_2_-RHSK NPs (white bars). (**d**–**f**) The release of inflammatory mediators IL-8 (**d**), IL-6 (**e**) and IL-1β (**f**) from the co-culture model of the lung exposed to LigNPs was assessed after 24 h of exposure to increasing concentrations (5 and 10 µg/cm^2^) of LigNPs (black bars) and PheLigNPs (dark grey bars). Data represents the mean ± SEM of three independent experiments (n = 3). * *p* < 0.05 compared to the control cells (ANOVA + Bonferroni’s test).

The effects of silica NPs (i.e., SiO_2_-Aerosil and SiO_2_-RHSK) and lignin-based NPs (i.e., LigNPs and PheLigNPs) were preliminarily tested in terms of cell viability and release of inflammatory mediators, also in an in vitro co-culture model of the lung. None of the NPs affected the cell viability of the co-culture model (Figure 7a). While in the monoculture model, the exposure to SiO_2_-Aerosil NPs significantly increased the release of ILs, in the co-culture model, the inflammatory response was not observed (Figure 7b,c). On the contrary, a reduction, though not significant, of IL-6 expression was observed (Figure 7c). With regards to LigNPs, data showed that in the co-culture model, PheLigNPs induced an increase in the release of IL-8 at both tested concentrations (5 and 10 µg/cm^2^, corresponding to the highest concentrations of 50 and 100 µg/mL used in the monocultures model) (Figure 7d), although not significant. IL-6 release was not influenced by the exposure to LigNPs (Figure 7e), while the exposure to PheLigNPs reduced the expression of IL-1β already at the concentration of 5 µg/cm^2^ and statistically significant at the concentration of 10 µg/cm^2^ (Figure 7f). The results of the positive control LPS (10 µg/mL) on the release of the three different cytokines from the co-culture is reported in the Supplementary Materials (Supplementary Figure S6).

## 4. Discussion

The concepts of sustainability, circularity and safety are three main pillars that need be considered for the further development of new chemicals and materials, including NMs and bio-based NMs, according to the safe and sustainable by design (SSbD) framework proposed by the JRC in 2022 [25]. In this perspective, the use and production of B-NMs derived from bio-based sources is largely increasing, due to the requests of the market to include sustainable technologies and to substitute as much as possible the use of petroleum-based composites in the production of materials, such as PU foams. This could be due by replacing a percentage of the oil-based products with natural-based composites—i.e., bio-polyols instead of conventional mineral oil-based polyols—and by adding chemical and materials derived from biomass wastes, used as (nano)fillers [49]. Moreover, the new chemicals, behind guaranteeing the same mechanical, thermal and conductive features of the conventional materials, may have enhanced properties, such as antimicrobial and flame-retardant activities. However, little is known about the impacts on both human and environmental health of B-NMs and their intrinsic hazard. The use of AOPs for the identification of biological endpoints to investigate the hazard of NMs, also called AOPs-oriented testing strategy, has been proposed as a valid method for the selection of the mechanisms of toxicity of NMs on the basis of the involved key events (KEs) [30]. This strategy and the use of in vitro methods, used as alternatives to animal testing in scientific research, are under the umbrella of new approaches methodologies (NAMs). NAMs are strongly promoted by the European Commission and are mentioned in the latest JRC document about the SSbD framework, also in accordance with the 3R (reduction, replacement and refinement) principle in animal research [25]. In this work, the AOP-testing strategy was used as reference to test specific biological endpoints that could be related to the onset of human diseases. Two different in vitro cells lines representative of the potential exposure routes to the B-NMs, namely the respiratory tract (A549) and the immune system (THP-1), were selected. A549 and THP-1 cells are commonly used as representative in vitro models of the lung tissue and immune cells and they have been extensively used for testing the toxicity of NMs. Moreover, since the use of in vitro models with a higher level of complexity and which more closely mimic the in vivo conditions are strongly recommended, an additional model representative of the alveolar space was considered: the co-culture of alveolar cells (A549) and THP-1–derived macrophages cultured at the air–liquid interface (ALI). The model was established at the ALI and exposure was performed in quasi-ALI. Then, according to an AOP-testing strategy, the assessment of the in vitro hazard potential of the different B-NMs, also in relation to their p-chem properties, was performed after identifying main MIE and KEs. According to the AOPs identified, the common KEs were selected, resulting in oxidative stress, inflammation and cytotoxicity. The experimental strategy was to assess these endpoints in the two cell lines exposed to the different Si- and LigNPs. Positive confirmation of the KEs, will support the potential of the tested B-NMs to induce the adverse outcome, while lack of the KEs activation would suggest a higher safety of the novel bio-based NMs.

Results showed that commercial fumed silica NPs (SiO_2_-Aerosil) were indeed able to trigger all the cited endpoints. SiO_2_-Aerosil was able to induce cell viability reduction, increased formation of intracellular ROS and increased release of IL-8 and IL-6 in both cell lines after 24 h of exposure. Significant increased release of IL-1β after exposure to SiO_2_-Aerosil was observed only in THP-1 cells. In fact, the expression of IL-1β is more related to immune cells, such as monocytes and macrophages, due to the activation of the inflammasome machinery [50]. The capability of Si-based particles to induce inflammation is not new. In vitro studies show that both micrometric and nanometric crystalline and non-crystalline particles may induce inflammation-related responses, including the release of proinflammatory cytokines (IL-6 and CXCL8) [51]. Since we report increased cell death at the highest concentration tested, the release of ILs at 100 µg/mL could also be a related indication from surrounding dying cells, which are able to activate a signalling pathway that stimulate the secretion of inflammatory mediators.

However, at the sub-cytotoxic concentration of 50 µg/mL, the release of IL-8 was increased by the exposure to SiO_2_-Aerosil NPs, suggesting that IL-8 is a good biomarker for investigating the inflammatory effects of SiNPs. The activation of the inflammatory response in exposed cells is indeed another key event (KE) in the AOP for lung fibrosis and dysfunction [41]. Our results are in accordance to what was already reported in the literature in which SiNPs induced cytotoxicity, inflammatory responses and increased oxidative stress in different cell lines, including lung and immune cells [26,52,53]. The generation of intracellular ROS induced by SiNPs has long been considered to be a dominating factor in their toxicity. However, it has also been reported that the induction of intracellular ROS depends on several NPs p-chem properties, such as size, surface, crystallinity, cell type and time of exposure [54,55]. Indeed, our data confirmed that intracellular ROS are induced by smaller NPs (SiO_2_-Aerosil) after 24 h of exposure, as previously observed also by Li and co-workers [56]. Significantly, the results showed that bio-based silica NPs (SiO_2_-RHSK) are less toxic, compared to commercial SiNPs, since no effects were observed in both A549 and THP-1 after the exposure to these NPs. Our results support the limited hazard posed by biogenic silica NPs, as previously reported [12,57].

SiNPs are known to induce toxicity in in vitro models depending on their size, agglomeration status, dose and surface modification [26]. The aggregation status of SiNPs plays an important role on the consequent biological effects, with aggregates of amorphous silica NPs of size higher that 100 nm being less toxic then the smaller counterparts [58]. Our results indeed showed that SiO_2_-RHSK NPs form big agglomerates once dispersed in mQ water, while SiO_2_-Aerosil NPs tend to form smaller agglomerates. However, when both NPs are dispersed in cell culture media, the NPs’ z-average highly increases, due to the interaction with the compounds of the medium. It is well known that the presence of serum proteins in cell culture media could lead to protein corona formation, influencing the z-average results, and consequently the interaction of NPs with the cell membrane [59]. However, it has also been reported that lowering the amount of foetal bovine serum (FBS) in the cell culture media to a final concentration of 1%, as performed in this work, could contribute in reducing the protein corona of nanoparticles [60].

In addition to the dimension of the agglomerates, the SiNPs synthesis process (wet—colloidal—versus pyrogenic synthesis) is known to affect the biological responses [61,62], since the p-chem characteristics of the resulting SiNPs are strictly related to the production process. Different studies evidenced that surface and shape modifications may mitigate the toxic effects of SiNPs, providing a means to produce NMs with lower toxic impacts. Our results support this knowledge since the introduction of functional groups such as PA and QAS or the grafting with polyols, affected differently the biological responses, suggesting that each new grafting should be tested separately to clearly determine its impact on the hazard properties of the NMs. In fact, the introduction of specific modifications, PA and QAS to SiO_2_-RHSK NPs highly changed the toxicity profiles of NPs. PA is a substance found in many plant-based foods and it is usually added to enhance the flame-retardancy properties of PU foam [50]. The safety and toxicity related to the exposure to flame retardants is a topic of great interest in recent years. We observed that the addition of PA + QAS to SiO_2_-RHSK highly increased their cytotoxicity, ROS formation and IL-8 release. This may be due to by-products formed during the synthesis process or to the sequestration by phytic acid and QAS (silane branching) of ions (Zn^+^, Fe^2+^) and nutrients from culture media fundamental for a cell’s survival [63]. Moreover, SiO_2_-RHSK + PA + QAS showed different p-chem characteristics compared to the other SiNPs, such as low hydrodynamic diameter and PdI values, as well as highly positive ζ-potential values (> +40 mV) that could be responsible for the higher cytotoxic and inflammatory effects observed after the exposure to these NPs. On the contrary, the addition of a polyol to the SiO_2_-RHSK NPs did not induce significant alteration of the effects in terms of cell viability and inflammation.

Regarding lignin-based NPs, there are few literature evidences about their hazard. Our results showed that both LigNPs and PheLigNPs induce increased ROS levels in both cell lines, while cytotoxicity was observed only in THP-1 cells at the highest tested concentrations. The induction of intracellular ROS generation, even after 90 min of exposure to both Lig and PheLig NPs, due to the presence of free radicals on their surface that interact with the cells, is also in accordance with what was previously reported by the literature on the effect of LigNPs in different cell lines [64]. Moreover, these findings agree with a previous study characterizing the balance between pro-oxidant and antioxidant effects of phenolic compounds. Despite phenolic compounds being widely recognized as antioxidants and used against oxidative stress, under certain environmental conditions (i.e., pH, presence of redox metals), they can exhibit pro-oxidant behaviour [65] and this property has been used for explaining the antibacterial effects of LigNPs and PheLigNPs. Moreover, the anti- and pro-oxidant effects of polyphenolic compounds could also depend on the target cell type. In cancer cells, polyphenols can lead to reduced levels of glutathione (GSH), due to their ability to exacerbate the formation of intracellular reactive oxygen species (ROS), which can induce redox imbalance and promote apoptotic or proliferative dysregulation of cancer abnormal cells [66]. In normal cells, the presence of polyphenols may help maintain GSH levels by reducing the damaging effects of ROS.

In previous works, the cytotoxicity of LigNPs was mainly assessed to investigate their safety as drug carriers or wound-healing agents. Alqahtani et al. [67] showed that the treatment with LigNPs could fasten the wound closure, while Siddiqui and colleagues [64] tested different formulation of LigNPs in different cell lines, including A549 cells, to ensure their compatibility with normal and cancer cells. The authors observed that bio-lignin NPs resulted to be safe on normal cells (HEK-293) and cytotoxic on cancer cells (A549 and MCF-7), but at very high concentrations (>500 µg/mL). The presence of folate receptor in MCF-7 cells could be responsible for the higher toxicity induced by LigNPs observed in cancer cells. Moreover, as previously mentioned, it has also been reported that pro-oxidant polyphenols reduce oxidative stress in normal cells and may induce apoptotic mechanisms in abnormal cells under exacerbating conditions of low GSH/GSSG ratio [66], facilitating the controlled activation of antitumoral drugs within cancer cells [59].

The biocompatibility in A549 cells of LigNPs, with different content of phenolic groups, was also demonstrated by Lee and co-workers, which demonstrated that LigNPs exhibited low cytotoxicity at concentrations of 25 and 50 μg/mL [68]. The biocompatibility of LigNPs has also been demonstrated on human keratinocytes (HaCat) cells until 7 days of exposure and in in vivo models [32]. With regards to lignin-based NPs’ inflammatory potential, we observed that PheLigNPs lowered the release of the inflammatory mediators IL-8 and Il-1β especially in A549 cell lines. The anti-inflammatory potential of green nanocomposites has been also demonstrated on human keratinocytes (HaCaT cells), with decreased expression of cytokines including IL-1α, IL-8 and TNF-α after the exposure to chitin nanofibril bio-lignin [65]. Moreover, due to the overall low release of this pro-inflammatory mediator, the data also suggest that A549 cells may not be the most suitable model for investigating IL-1β and further analyses are also needed in different cellular models. Nevertheless, the modulatory effects of LigNPs on the inflammatory response, especially on IL-8 release, are in accordance to what was also observed in *Danio rerio* embryos [23]. The effects of PheLigNPs in downregulating the levels of IL-8 was also previously observed in zebrafish embryos [23]. Moreover, it has also been reported that polyphenols are able to inhibit the production of IL-8 induced in response to an inflammatory event in both nasal epithelial cells and A549 cells [69]. Lack of activation of the inflammatory cascade after PheLigNPs, although their activation of ROS formation, suggests that these NPs could be promising for exploiting their antibacterial properties, in parallel to the possibility of preventing cytotoxicity in human cells.

According to the scientific community, the development of alternative methods and advanced in vitro models are essential for testing new chemicals and materials. In this perspective, an in vitro model of the alveolar space was used to assess the toxicity of NMs on a model representative of the respiratory epithelium. The use of the quasi-ALI model, although not as advanced as exposure at the ALI with specific exposure modules (i.e., Vitrocell Cloud-alpha, CULTEX-RFS), has been used to more closely mimic the in vivo situation. Moreover, the administration of a thin liquid layer may be also considered representative of what happens in the lungs, where a thin layer of surfactant covers the apical side of the cells. The results obtained from the exposure to SiNPs and LigNPs of the co-culture model showed a different response compared the results gained in monocultures, since no effect on cytotoxicity was observed. SiNPs also did not induce inflammatory responses in the co-culture model. Although the model here presented is a quasi-ALI model, it has previously been reported that SiNPs are less toxic when exposed at the ALI compared to submerged conditions. Panas and co-workers have indeed shown that the exposure of cells at the ALI to silica NPs induce less effects with respect to submerged co-cultures [70]. In the co-culture model, the ability of PheLigNPs to reduce IL-8 release as observed in the A549 monoculture models was not observed, probably due to the cross-talk among alveolar cells and macrophages in the co-culture model that modulates the release of ILs. However, not significant results were obtained for IL-8 release in the co-culture after exposure to both lignin-based NPs. Noteworthy, the use of the co-culture model better revealed the ability of PheLigNPs to reduce the release of IL-1β compared to LigNPs, since such a response was visible already at 10 µg/cm^2^ of exposure (Figure 7), likely due to the enrichment in the phenolic group of the LigNPs. Polyphenols, from a different origin, are indeed able to exert anti-inflammatory effects on different cell lines, including lung, intestinal and skin cells [69,71,72].

The different modulation in the inflammatory responses between the monoculture cell lines and the co-culture model could be due to the crosstalk between the different cells in the co-culture model and the capability of macrophagic cells receptors to uptake the cytokines released by alveolar cells, and vice versa. Also, the difference in the culturing and exposure procedure, submerged vs. quasi-ALI, may affect the outcome. The reported results support the importance to implement and use more complex in vitro models, composed by at least two different cell types, when performing hazard assessment of NMs, to have a more comprehensive interpretation of the results [40].

## 5. Conclusions

The investigation of the biological outcomes through an AOP-oriented approach, combined with the NPs characterization, allowed to estimate the cellular reactivity of the B-NMs, such as bio-based silica and lignin-based NPs. This approach is a useful tool for the first-tier screening of the profile of toxicity and hazards of newly synthetized B-NMs and gave new evidence about their biocompatibility in two different cell lines representative of the respiratory, the first route of exposure towards NMs, and immune systems. In vitro tests are fast, low-cost and mechanism-based alternatives to animal testing. The number of NMs from different sources and their sophisticated property variants requires the implementation of such tests to evaluate the safety of new materials in an SSbD perspective. Moreover, the combination with the AOP framework was useful to characterize the hazard of the different B-NMs by evidencing the KEs that could be involved in response to their exposure; in particular, the production and release of ROS and inflammatory mediators. Bio-based SiNPs resulted to be safer compared to commercial silica nanoforms that induce cytotoxicity and interleukins release. The introduction of specific modifications to SiNPs (PA + QAS) could increase the cytotoxic profile of bio-silica. However, the grafting of SiNPs from rice husk with bio-polyols did not affect their toxicity, allowing their safe incorporation in PU foams. Lignin NPs resulted to be safe in terms of cytotoxicity; indeed, cytotoxicity was observed in both tested cell lines and the co-culture model of the alveolar space only at the highest concentration of exposure. LigNPs were able to induce intracellular ROS increase and inflammatory potential. However, the presence of phenolic groups in PheLigNPs enhanced the antibacterial and oxidant properties of the B-NMs, without affecting their cytotoxicity and by promoting the reduction in the inflammatory mediators IL-8 and IL-6. Altogether, results showed that attention should be given to possible AOs deriving after specific functionalization of the B-NMs (by i.e., addition of phenolic groups or phytic acid) that determined cytotoxic, inflammatory and pro-oxidative effects. This evidence suggests that an SSbD approach should be applied also after specific functionalization of originally non-toxic B-NMs to produce efficient new materials, while minimizing their hazardous impacts. Considering the lack of knowledge in this field, the nanotoxicity studies performed in this work represent a step forward in the state of the art of the safety assessment of novel B-NMs. Future perspectives are focused on the implementation of omics (transcriptomics) approaches in the study of AOPs and a more relevant in vitro model of exposure to realistic doses of B-NMs, following an integrated new approach methodology that combines the data from the field monitoring campaign during the synthesis of B-NMs or their incorporation in PU foams, deposition modelling and in vitro exposure directly at the ALI through exposure modules. Future research should also be focused on the assessment of long-term effects of B-NMs and their potential impacts on the environment. Moreover, the real-world applications of B-NMs would need to be further investigated, as well as the hazard assessment of B-NMs at other phases of their life cycle, such as during the manufacturing phase, when they are incorporated in the nano-enabled products (i.e., PUR foams) or at their use phase, when B-NMs could potentially be released from the final products into the environment.

## Figures and Tables

**Table 1:** B-NMs p-chem characterization. Dynamic light scattering (DLS) analyses were performed for SiNPs and LigNPs in milli-Q (mQ) water, and in two cell culture media: OptiMEM and DMEM, both supplemented with 1% FBS. The measurements were performed at concentrations of 100 µg/mL. In the table are also reported the values of z-average (nm) ± SD and Polidispersity Index (PDI) ± SD. In addition, for each particle is indicated the value ζ-potential (mV) ± SD in milli-Q water measured at the concentration of 100 µg/mL by ELS.

NPs (100 µg/L)	Medium	Time (h)	Zeta-Average (nm) ± SD	PdI ± SD	ζ-Potential (mV)
**SiO_2_-Aerosil**	**mQ water**	0	246.7 ± 40.5	0.225 ± 0.0002	−21.03 ± 0.62
	**mQ water**	24	218.7 ± 20.7	0.225 ± 0.022	
	**DMEM 1% FBS**	0	625.5 ± 344.7	0.59 ± 0.23	
	**DMEM 1% FBS**	24	185.7 ± 4.12	0.168 ± 0.068	
	**Opti-MEM 1% FBS**	0	702.1 ± 261.6	0.588 ± 0.12	
	**Opti-MEM 1% FBS**	24	629.1 ± 190.6	0.771 ± 0.139	
**SiO_2_-RHSK**	**mQ water**	0	507. 9 ± 104.8	0.693 ± 0.037	−22.80 ± 0.14
	**mQ water**	24	726.9 ± 208.66	0.818 ± 0.052	
	**DMEM 1% FBS**	0	1022.7 ± 510.04	0.888 ± 0.096	
	**DMEM 1% FBS**	24	154.9 ± 62.52	0.226 ± 0.241	
	**Opti-MEM 1% FBS**	0	1359.1 ± 821.5	0.925 ± 0.110	
	**Opti-MEM 1% FBS**	24	177.9 ± 29.6	0.296 ± 0.166	
**LigNPs**	**mQ water**	0	355.5 ± 7.366	0.197 ± 0.017	−33.2 ± 2.5
	**mQ water**	24	293.0 ± 4.456	0.172 ± 0.043	
	**DMEM 1% FBS**	0	528.8 ± 2.41	0.162 ± 0.122	
	**DMEM 1% FBS**	24	210.9 ± 4.15	0.16 ± 0.011	
	**Opti-MEM 1% FBS**	0	533.3 ± 9,00	0.241 ± 0.006	
	**Opti-MEM 1% FBS**	24	366.1 ± 5.16	0.103 ± 0.094	
**PheLigNPs**	**mQ water**	0	525.4 ± 23.290	0.345 ± 0.016	−32.7 ± 0.451
	**mQ water**	24	568.9 ± 10.060	0.099 ± 0.015	
	**DMEM 1% FBS**	0	871.2 ± 107.1	0.367 ± 0.08	
	**DMEM 1% FBS**	24	196.6 ± 4.64	0.113 ± 0.046	
	**Opti-MEM 1% FBS**	0	918.7 ± 42.48	0.398 ± 0.103	
	**Opti-MEM 1% FBS**	24	396.6 ± 11.28	0.262 ± 0.011	

## Data Availability

Data are available from the researchers on request.

## Supplementary Materials

The following supporting information can be downloaded at: https://www.mdpi.com/article/10.3390/nano15070549/s1. **Figure S1**: P-chem characterization of SiNPs derived from rice husk (SiO_2_-RHSK) and commercially available particles (SiO_2_-Aerosil): TG and DTG (a – for SiO_2_-RHSK); FTIR spectra (c); SEM-EDS data (for SiO_2_-RHSK only. The inserts in the upper right part show photographs of the analysed sample); **Figure S2.** TEM images of SiNPs modifications: (a) TEM image of SiO_2_-RHSK-OH NPs; (b) TEM image of PM134 NPs (the suspension was prepared in DMSO). Scale bars = 0.5 µm. **Table S1**. SiNPs modifications characterization. Dynamic Light Scattering (DLS) analysis performed in MilliQ water (mQ) or DMEM 1% FBS. The measurements were performed at concentrations of 100 µg/mL. In the table are reported the values of z-average (nm) ± SD and PDI ± SD. In addition, for each particle is indicated the value ζ-potential (mV) in mQ water at the concentration of 100 µg/mL, when measured; **Figure S3.** A549 responses to SiNPs modifications. (a) Cell viability of A549 cells exposed for 24 h to commercial fumed SiNPs (SiO_2_-Aerosil) and biomass based NMs (RHSK) and their modification with functional groups. OH: hydroxyl group; PA: phytic acid; QAS: 3-(trimethoxysilyl)-propyldimethyloctadecylammonium chloride. (b) ROS level in A549 cells exposed for 24h to 50 µg/mL of SiO_2_-Aerosil, SiO_2_-RHSK, SiO_2_-RHSK-OH and SiO_2_-RHSK+PA+QAS NPs. c) IL-8 release from A549 after exposure to SiNPs modifications. Data represents the mean over control ± SEM of 3 independent experiments (n = 3). * *p* < 0.05; *** *p* < 0.001 (One-Way ANOVA + Bonferroni’s test). **Figure S4.** Effect of SiNPs grafted with polyol (BI-3521) in A549 cells. Cell viability (a) and IL-8 release (b) of A549 cells exposed for 24 h to SiO_2_-RHSK NPs and their modification by adding a commercial polyol (PM134). Moreover, both cell viability and IL-8 was assessed after the exposure to the amount of DMSO used for the preparation of NPs suspensions, tested at the different concentrations (DMSO eq). Data represents the mean ± SEM of at 3 independent experiments (n = 3). * *p* < 0.05 (One-Way ANOVA + Dunn’s test). **Figure S5**. ROS formation after exposure to positive control. The level of intracellular ROS in both A549 and THP-1 cells were measured after 90 min of exposure to 100 µm of H_2_O_2_. Data represent the mean ± SEM of fold change respect to control cells of at least three independent experiments (n ≥ 3). * *p* <0.05 (Kruskal-Wallis One-Way ANOVA on Ranks + Dunn’s test). **Figure S6.** Release of IL-8 (a), IL-6 (b) and IL-1β from (c) A549 and THP-1 cells and co-culture after exposure to the positive control LPS (10 µg/mL) for 24h. Data represent the mean ± SEM of fold change respect to control cells of at least three independent experiments (n ≥ 3). ** *p* < 0.01; *** *p* < 0.001 (One-Way ANOVA + Bonferroni’s test).

## Appendix A

List of adverse outcome pathways (AOPs)

**Table A1:** List of AOPs (from AOP-Wiki) which have as stressors nanoparticles, silica NPs, insoluble nanosized particles. In bold are highlighted the key events (KEs) that are common for almost all the AOPs here shown and that have been selected as biological endpoints for the hazard assessment of the B-NMs.

Stressor (ID: Title)	AOP (ID: Title)	KEs (ID: Title)	AO (IDr: Title)
**224: nanoparticles**	144: Endocytic lysosomal uptake leading to liver fibrosis	898: Disruption, Lysosome 177: Mitochondrial dysfunction **55: Cell injury/death** **1493: Increased Pro-inflammatory mediators** 1494: Leukocyte recruitment/activation 265: Activation, Stellate cells 68: Accumulation, Collagen	344: Liver Fibrosis
	392: Decreased fibrinolysis and activated bradykinin system leading to hyperinflammation	**1496: Increased, secretion of proinflammatory mediators** **1497: Increased, recruitment of inflammatory cells**	1868: Hyperinflammation
	451: Interaction with lung resident cell membrane components leads to lung cancer	**1496: Increased, secretion of proinflammatory mediators** **1497: Increased, recruitment of inflammatory cells** **780: Increase, Cytotoxicity (epithelial cells)** **1115: Increased, Reactive oxygen species** 2006: Secondary genotoxicity 1669: Increased, DNA damage and mutation 870: Increase, Cell Proliferation Increase	1670: Lung cancer
**254: silica nanoparticles**	209: Perturbation of cholesterol and glutathione homeostasis leading to hepatotoxicity: Integrated multi-OMICS approach for building AOP	1284: Up Regulation, SREBF2 1285: Up Regulation, Unsaturated fatty acid Up Regulation, 1286: Down Regulation, GSS and GSTs gene 1287: Glutathione synthesis 1288: Activation, 3-hydroxy-3-methylglutaryl-CoA reductase gene 1289: Perturbation of cholesterol 1290: Glutathione homeostasis	1291: Hepatoxicity
	481: AOPs of amorphous silica nanoparticles: ROS-mediated oxidative stress increased respiratory dysfunction and diseases.	**1392: Oxidative Stress;** 1816: Mitochondrial dysfunction; **1586: Airway epithelial injury ** **1198: Activation, Macrophages** **2085: Pulmonary epithelial injury** 1197: Activation, Fibroblasts **2086: Airway inflammation** **2010: Pulmonary inflammation** 1458: Pulmonary fibrosis	2087: Increased incidence of respiratory disease 2088—Respiratory dysfunction
**377: Insoluble nanosized particles**	237: Substance interaction with lung resident cell membrane components leading to atherosclerosis	**1496: Increased, secretion of proinflammatory mediators** 1438: Transcription of genes encoding acute phase proteins, Increased 1439: Systemic acute phase response	1443: Atherosclerosis
