# Supplementary material for: Adverse Outcome Pathways (AOPs) Oriented Approach to Assess In Vitro Hazard of Silica and Lignin Nanomaterials Derived from Biomass Residues
Source: Nanomaterials (Basel). 2025 Apr 4;15(7):549. doi: 10.3390/nano15070549 (PMC11990304; doi:10.3390/nano15070549)
Supplement: Supplementary file 1 [file nanomaterials-15-00549-s001.zip › nanomaterials-3511508-supplementary.pdf]

Adverse outcome pathways (AOPs) oriented approach to assess *in vitro* hazard of silica and lignin nanomaterials derived from biomass residues.

Rossella Daniela Bengalli*[1,2], Maurizio Gualtieri*[1], Mariana Ornelas[3], Tzanko Tzanov[4], Paride Mantecca[1,2]

[1] POLARIS Research Center, Department of Earth and Environmental Sciences, University of Milano-Bicocca, Piazza della Scienza 1, 20126 Milan, Italy

[2] Interuniversity Center for the Promotion of the 3Rs Principles in Teaching and Research, Italy

[3] CeNTI - Centre for Nanotechnology and Advanced Materials, Rua Fernando Mesquita 2785, 4760-034 V. N. Famalicão, Portugal

[4] Group of Molecular and Industrial Biotechnology, Universitat Politècnica de Catalunya, Terrassa, 08222, Spain

* Correspondence: rossella.bengalli@unimib.it (R.B.), maurizio.gualtieri@unimib.it (M.G.)

Supplementary materials

1. SiNPs characterization: Thermogravimetric analysis, FTIR and SEM-EDS

FTIR-ATR analysis was performed using a Perkin Elmer Spectrum 100 Series spectrophotometer with a spectral range and resolution factor of 4000 to 650 cm^{-1} and 8 cm^{-1}, respectively. The morphology and chemical composition of the particles were characterized by scanning electron microscopy (SEM) connected to an energy dispersive X-ray spectroscopy (EDS) using a NanoSEM – FEI Nova 200 (FEG/SEM) and EDAX - Pegasus X4M (EDS/EBSD) with high vacuum resolution 1.8 nm at 1 kV (SE) 1.0 nm at 15 kV (SE) or low vacuum resolution 1.8 nm at 3 kV (Helix detector) 1.5 nm at 10 kV (Helix detector). The electron beam resolution of 0.8 nm at 30 kV (STEM), beam landing energy: 200V to 30 kV, high stability Schottky field emission gun with automatic operation, probe current: 0.3 pA to 22 nA, chamber vacuum (high vacuum): <10-4 mBar and chamber vacuum (low vacuum): <2 mBar were selected as the optimal operation conditions.

The thermogravimetric analysis (TGA) was performed on a TG 209 F1 Libra apparatus (Netzsch) with a Top-loading type of thermobalance. The test was carried out by an internal method “Evaluation of the thermal stability of materials by thermogravimetry” based on the standard ISO 11358:1997(E). The samples were pre-conditioned at laboratory's ambient conditions: room registered temperature (≈ 23 °C) and relative humidity (≈ 51%). For the sample pre-treatment, each one was properly separated, in order to fit inside the crucible (volume of 85 µL) and to ensure maximum contact with its bottom. The tests were accomplished in a synthetic air atmosphere, following a temperature program from 30 to 700 °C, with a heating rate of 20 K/min. In the supplementary figure S1 are reported the further p-chemical characterization evaluated through TG and DTG (Figure S1a, b), FTIR (c), and SEM-EDS analyses (d) for the bio-based silica SiO_2-RHSK. The results are commented in the main text.

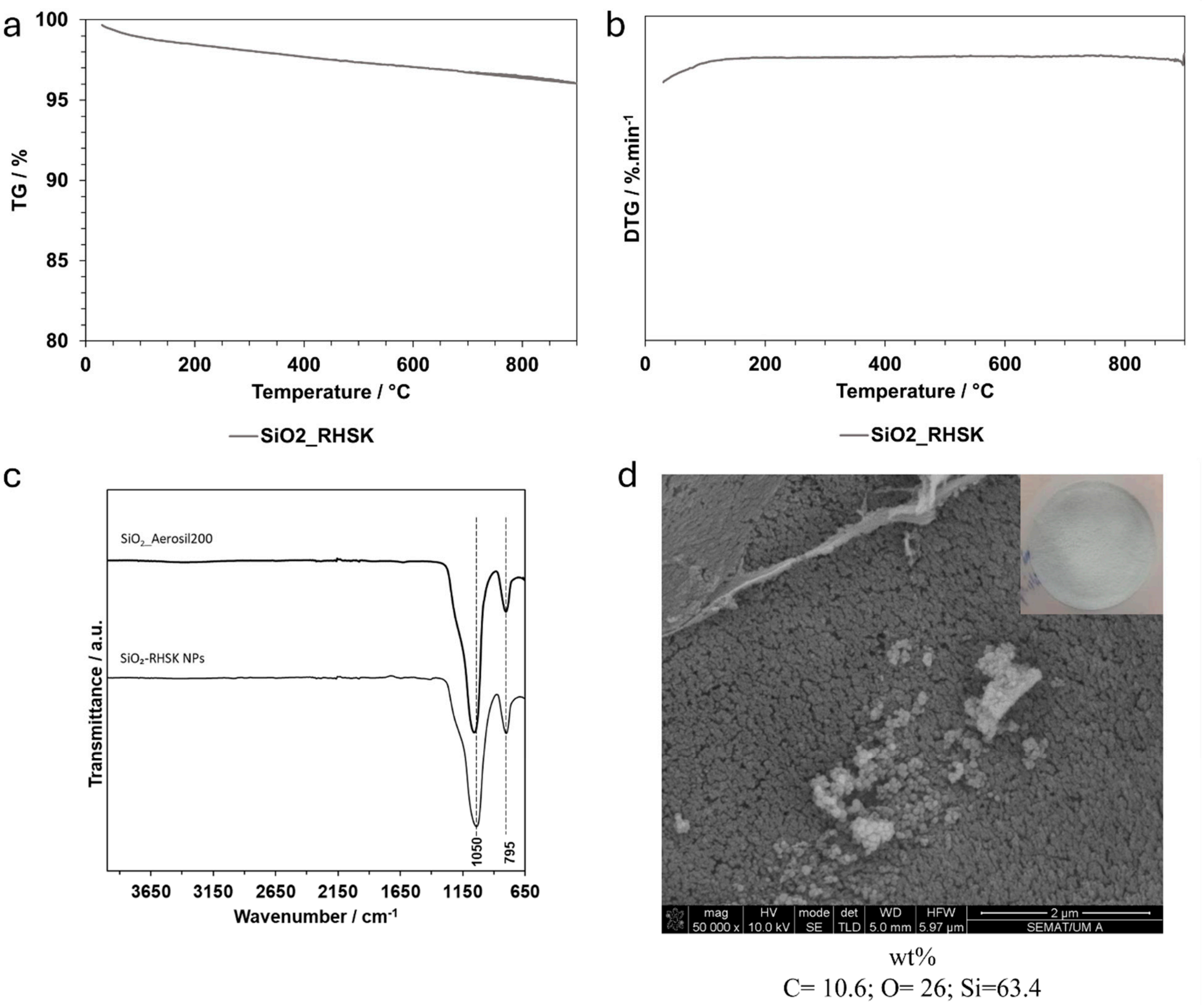

Supplementary Figure S1. P-chem characterization of SiNPs derived from rice husk (SiO_2-RHSK) and commercially available particles (SiO_2-Aerosil): TG and DTG (a – for SiO_2-RHSK); FTIR spectra (c); SEM-EDS data (for SiO_2-RHSK only. The inserts in the upper right part show photographs of the analysed sample).

2.1 SiNPs modifications: synthesis

SiO_2-RHSK NPs were used as basis for the synthesis of functionalized NPs:

1. SiO_2-RHSK NPs with hydroxyl groups (SiO_2-RHSK-OH)
2. SiO_2-RHSK with phytic acid (PA) and (3-(trimethoxysilyl)-propyldimethyloctadecylammonium chloride (QAS) (SiO_2-RHSK+PA+QAS);
3. SiO_2-RHSK NPs grafted with polyol (BI-3521), called PM134. BI-3521 is a polyol derived from a two-step process, consisting on the epoxidation of plant oils and the successive ring opening of the epoxy groups. It is a REACH polymer, with an OH value of 367 mg KOH/g, a viscosity of 1.209 cPs and a calculated bio-based content of 48,5 % wt.

For synthetizing SiO_2-RHSK+PA+QAS NPs, SiO_2-RHSK was dispersed in EtOH:H_2O (1:1) and phytic acid (PA) was added to this mixture being sonicated for 30 min. Then, 3-(trimethoxysilyl)-propyldimethyloctadecylammonium chloride (QAS) was added dropwise to the previous mixture and the reaction was left during 24h at ≈ 22 °C. At the end, a centrifugation step under 9000 rpm (10 min)

was performed and several washing actions (three times) were completed. The granulates obtained were dried at 40 °C and grinded to obtain the desired powder to further studies.
PM134 NMs were resuspended in DMSO to obtain a better dispersion, due to the hydrophobicity of polyols in aqueous media.

2.2 SiNPs modifications: p-chem characterization

SiNPs modified with functional groups were characterized at TEM and through DLS. Data from TEM analysis showed that SiO_2-RHSK-OH NPs for big agglomerates, as well as SiO_2-RHSK+PA+QAS NPs (data not showed), while images from PM134 (silica NPs grafted with polyol) showed that these NPs are spherical, have a core size of around 500 nm and several spikes on the surface. DLS analyses (Table S1) showed SiO_2-RHSK-OH NPs have a z-average of 538.87 ± 126.88 and negative ζ-potential (-30.9 ± 0.4 mV), while SiO_2-RHSK+PA+QAS have a mean hydrodynamic diameter of 277 ± 9 nm and positive surface charge (+41.0 ± 2.2 mV). PM134 was analysed in DMEM 1% and DLS data showed that the z-average of this NP is 219 ± 132 nm.

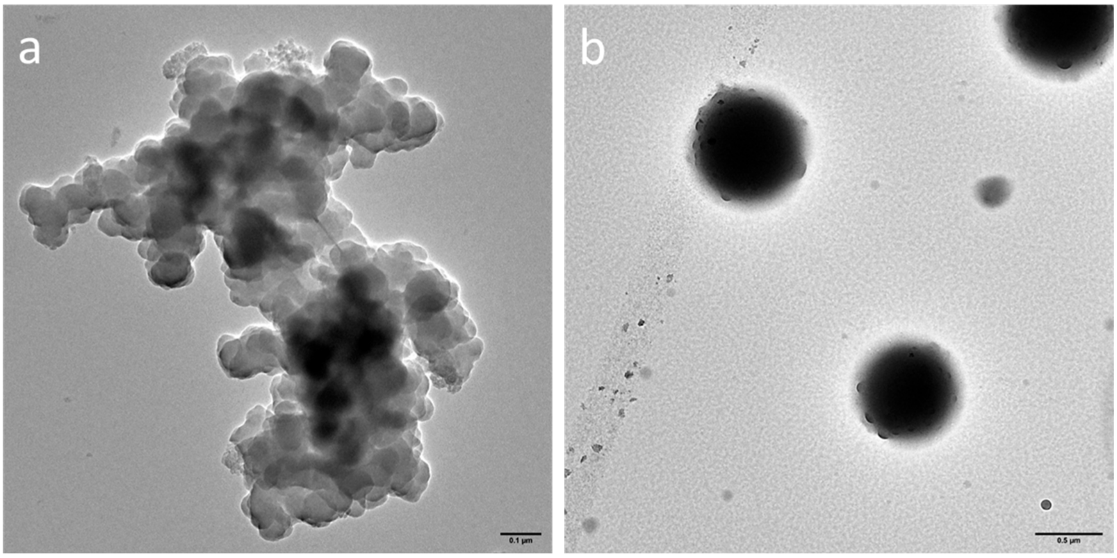

Supplementary Figure S2. TEM images of SiNPs modifications: a) TEM image of SiO2-RHSK-OH NPs; b) TEM image of PM134 NPs (the suspension was prepared in DMSO). Scale bars= 0.5 µm.

Table S1. SiNPs modifications characterization. Dynamic Light Scattering (DLS) analysis performed in MilliQ water (mQ) or DMEM 1% FBS. The measurements were performed at concentrations of 100 µg/mL. In the table are reported the values of z-average (nm) ± SD and PDI ± SD. In addition, for each particle is indicated the value ζ-potential (mV) in mQ water at the concentration of 100 µg/mL, when measured.

Sample	Medium	z-average (nm)	PdI	ζ-potential (mV)
**SiO_2-RHSK-OH**	mQ	538.87 ± 126.88	0.65 ± 0.13	-30.9 ± 0.4
**SiO_2-RHSK+PA+QAS**	mQ	277 ± 9	0.222	41.0 ± 2.2
**PM134**	DMEM	219 ± 132	0.41 ± 0.15	n.d.

2.3 SiNPs modifications: *in vitro* hazard assessment in A549 cells

Since the biological effects induced by commercial SiO_2-Aerosil NPs and SiO_2-RHSK NPs were similar in A549 cells and THP-1 cells, the test with functionalized SiNPs (SiO_2-RHKS-OH and SiO_2-RHSK+PA+QAS) were performed only on A549 cells. Cytotoxicity, ROS production and the release of IL-8 were investigated. Cell viability data showed that SiO_2-RHSK+PA+QAS induced a significant cell death in A549 cells exposed to 100 µg/mL of NPs, comparable to the one observed to SiO_2-Aerosil reference NPs (Supplementary Figure S3a). Moreover, ROS levels and IL-8 release resulted increased after 24h of exposure to SiO_2-RHSK+PA+QAS (Supplementary Figure S3b and c).

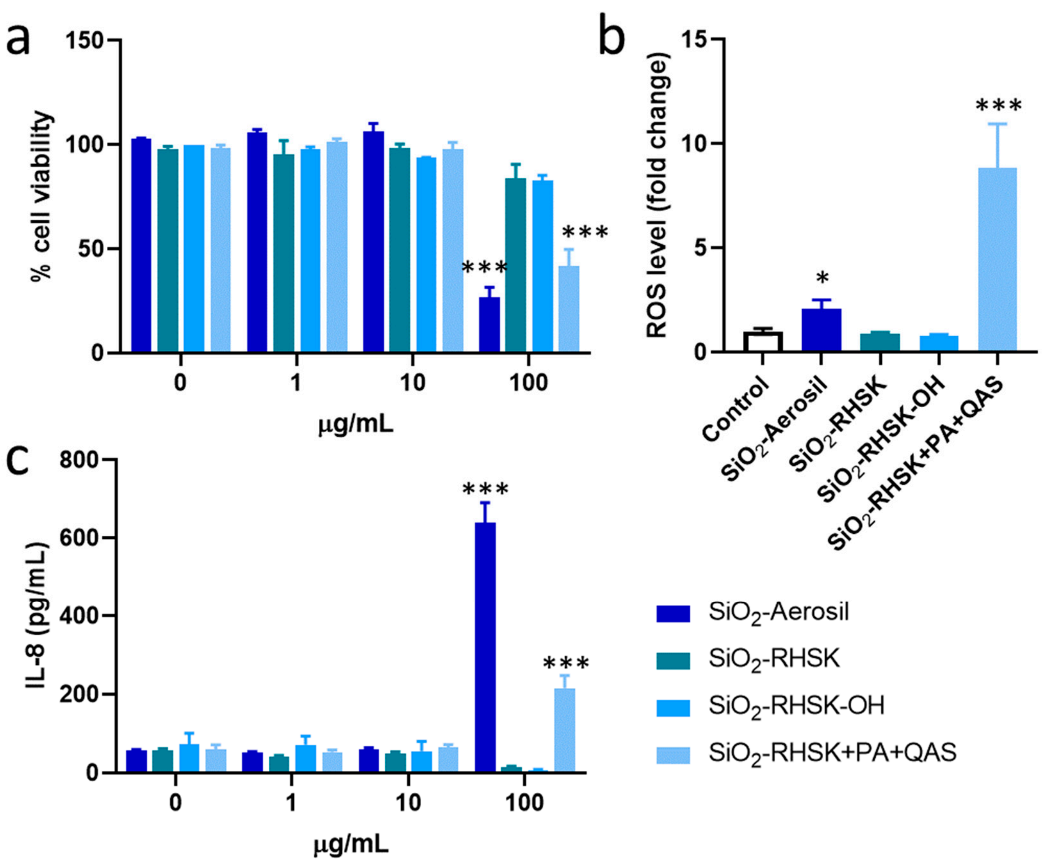

Supplementary Figure S3. A549 responses to SiNPs modifications. a) Cell viability of A549 cells exposed for 24 h to commercial fumed SiNPs (SiO_2-Aerosil) and biomass based NMs (RHSK) and their modification with functional groups. OH: hydroxyl group; PA: phytic acid; QAS: 3-(trimethoxysilyl)-propyldimethyloctadecylammonium chloride. b) ROS level in A549 cells exposed for 24h to 50 µg/mL of SiO_2-Aerosil, SiO_2-RHSK, SiO_2 RHSK-OH and SiO_2 RHSK+PA+QAS NPs. c) IL-8 release from A549 after exposure to SiNPs modifications. Data represents the mean over control ± SEM of 3 independent experiments (n=3). *$p<0.05$; ***$p<0.001$ (One-Way ANOVA + Bonferroni's test).

The impacts of SiO_2-RHSK grafted with commercial polyol BI-3521 (SiNPs called PM134) was also studied in terms of cytotoxicity and release of IL-8 in A549. Data showed that the grafting did not

influenced A549 cell viability and the inflammatory response, suggesting that the compound here investigated is not cytotoxic for the lung epithelium (Supplementary Figure S4).

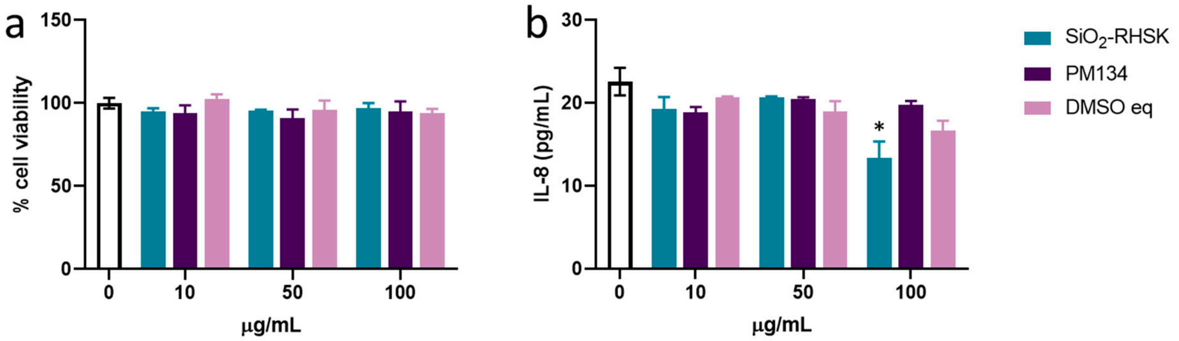

Supplementary Figure S4. Effect of SiNPs grafted with polyol (BI-3521) in A549 cells. Cell viability (a) and IL-8 release (b) of A549 cells exposed for 24 h to SiO_2-RHSK NPs and their modification by adding a commercial polyol (PM134). Moreover, both cell viability and IL-8 was assessed after the exposure to the amount of DMSO used for the preparation of NPs suspensions, tested at the different concentrations (DMSO eq). Data represents the mean ± SEM of at 3 independent experiments (n=3). *p<0.05 (One-Way ANOVA + Dunn's test).

3. ROS formation and inflammatory responses induced by positive controls

Results obtained by the exposure to A549 and THP-1 cells to positive controls, respectively H_2O_2 (100 μM) for 90 min for ROS formation and LPS (10 μg/mL) for the induction of inflammatory cytokines, are shown in supplementary Fig. S5. Data showed that H_2O_2 is able to induce a significant increase of intracellular ROS compared to control cells of around 5-fold in A549 cells and of 2.6-fold in THP-1 cells (Supplementary Figure 5).

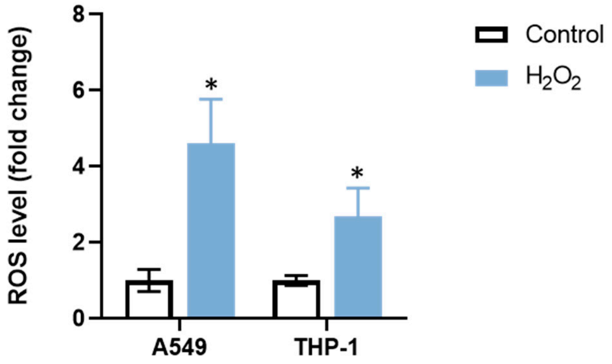

Supplementary Figure S5. ROS formation after exposure to positive control. The level of intracellular ROS in both A549 and THP-1 cells were measured after 90min of exposure to 100 Um of H2O2. Data represent the mean ± SEM of fold change respect to control cells of at least three independent experiments (n≥3). *p <0.05 (Kruskal-Wallis One-Way ANOVA on Ranks + Dunn's test).

LPS (10 μg/mL, corresponding to 1 μg/cm^2) was used as positive control for the induction of the release of inflammatory mediators from A549 and THP-1 cells and co-culture. Data (reported in

Supplementary Figure S6) show that LPS induces significant release of IL-8 in both A549 and THP-1 cells, release of IL-6 and IL-1β from THP-1 cells. No release of cytokines from the co-culture system was observed at the tested concentration, although previous works have demonstrated that a concentration of LPS of 10-fold higher (10.42 μg/cm^2) induce the release of all three cytokines in the same co-culture *in vitro* model (Motta et al. 2024, ref [37]).

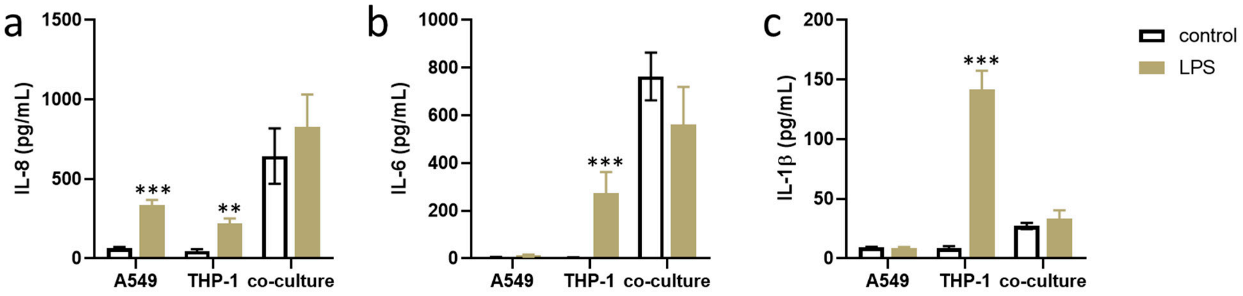

Supplementary Figure S6. Release of IL-8 (a), IL-6 (b) and IL-1β from (c) A549 and THP-1 cells and co-culture after exposure to the positive control LPS (10 μg/mL) for 24h. Data represent the mean ± SEM of fold change respect to control cells of at least three independent experiments (n≥3). **p <0.01; ***p<0.001 (One-Way ANOVA + Bonferroni's test).
